# Candidate Gene Analysis Reveals That the Fruit Color Locus *C1* Corresponds to *PRR2* in Pepper (*Capsicum frutescens*)

**DOI:** 10.3389/fpls.2020.00399

**Published:** 2020-04-09

**Authors:** Hyo-Bong Jeong, So-Jeong Jang, Min-Young Kang, Suna Kim, Jin-Kyung Kwon, Byoung-Cheorl Kang

**Affiliations:** ^1^Laboratory of Horticultural Crops Breeding & Genetics, Department of Plant Science, Plant Genomics and Breeding Institute, and Research Institute of Agriculture and Life Sciences, Seoul National University, Seoul, South Korea; ^2^Food and Nutrition in Home Economics, Korea National Open University, Seoul, South Korea; ^3^Institutes of Green Bio Science and Technology, Seoul National University, Seoul, South Korea

**Keywords:** mature fruit color, pepper (*Capsicum* spp.), carotenoid, plastid, *Pseudo response regulator2-like* gene

## Abstract

The diverse fruit colors of peppers (*Capsicum* spp.) are due to variations in carotenoid composition and content. Mature fruit color in peppers is regulated by three independent loci, *C1*, *C2*, and *Y*. *C2* and *Y* encode phytoene synthase (PSY1) and capsanthin-capsorubin synthase (CCS), respectively; however, the identity of the *C1* gene has been unknown. With the aim of identifying *C1*, we analyzed two pepper accessions with different fruit colors: *Capsicum frutescens* AC08-045 and AC08-201, whose fruits are light yellow and white, respectively. Ultra-performance liquid chromatography showed that the total carotenoid content was six times higher in AC08-045 than in AC08-201 fruits, with similar composition of main carotenoids and slight difference in minor components. These results suggest that a genetic factor in AC08-201 may down-regulate overall carotenoid biosynthesis. Analyses of candidate genes related to carotenoid biosynthesis and plastid abundance revealed that both accessions carry non-functional alleles of *CCS*, *golden2-like transcription factor* (*GLK2*), and *PSY1.* However, a nonsense mutation (C2571T) in *PRR2*, a homolog of *Arabidopsis pseudo response regulator2-like* (*APRR2*), was present in only AC08-201. In a population derived from a cross between AC08-045 and AC08-201, a SNP marker based on the nonsense mutation co-segregated fully with fruit color, implying that the mutation in *PRR2* may cause the white color of AC08-201 fruits. Transmission electron microscopy (TEM) of AC08-201 fruit pericarp also showed less developed granum structure in chloroplast and smaller plastoglobule in chromoplast compared to those of AC08-045. Virus-induced gene silencing (VIGS) of *PRR2* significantly reduced carotenoid accumulation in *Capsicum annuum* ‘Micropep Yellow’, which carries non-functional mutations in both *PSY1* and *CCS*. Furthermore, sequence analysis of *PSY1*, *CCS*, and *PRR2* in other white pepper accessions of *C. annuum* and *Capsicum chinense* showed that they commonly have non-functional alleles in *PSY1*, *CCS*, and *PRR2*. Thus, our data demonstrate that the fruit color locus *C1* in *Capsicum* spp. corresponds to the gene *PRR2*.

## Introduction

Carotenoids, also called tetraterpenoids, are derived from 8 isoprene units and contain 40 carbons in their polyene backbone. Carotenoids are essential for human nutrition and health as they provide dietary sources of provitamin A and serve as antioxidants that reduce the incidence of many diseases, including age-related eye diseases, cardiovascular diseases, and cancers (Fraser and Bramley, 2004). Carotenoids also play crucial roles in plants, including photosynthesis and photoprotection, and provide precursors for the biosynthesis of phytohormones such as abscisic acid (ABA) and strigolactones (Domonkos et al., 2013; Nambara and Marion-Poll, 2005; Al-Babili and Bouwmeester, 2015). The carotenoid biosynthetic pathway and its associated enzymes have been well elucidated in Solanaceous plants. Carotenoid biosynthesis begins with the formation of phytoene with the aid of phytoene synthase (PSY). Through a series of desaturation and isomerization steps, phytoene is converted into lycopene, which is the major carotenoid component in mature tomato (*Solanum lycopersicum*). Whereas the red color in tomato is due to lycopene accumulation, the red color in peppers results from the accumulation of capsanthin and capsorubin (Paran and van der Knaap, 2007). This pepper-specific process is regulated by capsanthin-capsorubin synthase (CCS), which converts downstream products of lycopene, antheraxanthin and violaxanthin into capsanthin and capsorubin, respectively (Bouvier et al., 1994).

The distinct colors of mature pepper fruits result from the conversion of chloroplasts to chromoplasts during ripening. Compared to chloroplasts, chromoplasts have a greater capacity to synthesize and sequester carotenoids, and as a result the fruits change from their immature green to display ivory to red colors in maturity (Sun et al., 2018). Based on inheritance studies of pepper fruit color, a three-locus model (*C1*, *C2*, and *Y*) was proposed by Hurtado-Hernandez and Smith (1985). In this model, mature fruit color can be classified into eight groups according to their allelic combinations, ranging from white (*c1 c2 y*) to red (*C1 C2 Y*). Candidate gene analyses of carotenoid biosynthetic genes revealed that the *C2* and *Y* loci are the sites of the genes *PSY1* and *CCS*, respectively (Popovsky and Paran, 2000; Huh et al., 2001; Lefebvre et al., 1998), but the nature of the third locus, *C1*, remained to be discovered. Since the identification of the *PSY1* and *CCS* genes, many researchers have tried to understand fruit-color variation in peppers based on the allelic variations of these two loci. However, since fruit color is not only qualitative, but can also be quantitative, explanations of fruit-color variation based on only *PSY1* and *CCS* are limited.

Other researchers have analyzed quantitative variation in color intensity in Solanaceous fruits. Brand et al. (2012) revealed that the chlorophyll content of immature pepper fruits is mainly controlled by two quantitative trait loci (QTLs) designated *pc8* and *pc10*, respectively. *pc10* encodes the golden2-like transcription factor (GLK2), which regulates chloroplast compartment size during the early stages of fruit development (Brand et al., 2014). The identity of *pc8* had not been known, although several transcription factor genes have been regarded as candidates, including *Arabidopsis pseudo response regulator2-like* (*APRR2*), as the gene of *pc8* (Brand et al., 2014). Only recently, it was identified as the pepper homolog of transcription factor *LSD ONE LIKE1* (*LOL1*), which eventually termed *pc1* as further mapping showed the QTL in chromosome 1. However, mutation of *CcLOL1* showed significant difference in fruit chlorophyll, but not in carotenoid contents (Borovsky et al., 2019). Similar to the case of *CcLOL1* mutant pepper, one *C. annuum* pepper cultivar that is white when immature, a nonsense mutation in *APRR2* is strongly associated with color intensity in the immature fruits (Pan et al., 2013). Although both *GLK2* and *APRR2* affect the color of immature pepper fruits, overexpression of each gene in tomato led to increased levels of carotenoids as well as chlorophyll (Powell et al., 2012; Pan et al., 2013). By contrast, the possible involvement of *GLK2* and *APRR2* in the color of mature pepper fruits was unknown.

In this study, we performed a candidate analysis to identify the gene in the *C1* locus that controls white fruit color. Six carotenoid biosynthetic genes along with two plastid related genes were analyzed. We showed that the white color of *C. frutescens* AC08-201 is due to a nonsense mutation in *PRR2*, as well as *PSY1* and *CCS* mutations. Expression analysis of *PRR2* showed that this gene is specifically expressed at the immature fruit stage. A genetic analysis demonstrated that the nonsense mutation co-segregated fully with the fruit color in a population derived from a cross between AC08-045 and AC08-201. Virus-induced gene silencing of the *PRR2* gene of *C. annuum* cultivar MY demonstrated that down-regulation of *PRR2* resulted in lighter color of both immature and mature fruits. Furthermore, sequence analysis of *PRR2* showed that two additional white pepper cultivars, from *C. annuum* and *C. chinense*, also had non-functional alleles in *PSY1*, *CCS*, and *PRR2*. In conclusion, we have demonstrated that *PRR2* is the gene corresponding to *C1*.

## Materials and Methods

### Plant Materials

*Capsicum frutescens* accessions AC08-045 and AC08-201 were selected from the germplasm of the Horticultural Crops Breeding and Genetics lab (Seoul National University, South Korea). Both accessions have bush-type architecture and set numerous fruit. A total of 127 F_2_ individuals were obtained from a cross between AC08-045 and AC08-201, and this population was grown in the experimental greenhouse of Seoul National University (Suwon, South Korea). Dominance of light yellow over white fruit color was confirmed in an F_1_ hybrid.

*Capsicum annuum* ‘MicroPep Yellow (MY)’ was used for VIGS analysis because of its fast growth rate and short internode length. MY peppers have dark green immature fruits and yellow mature fruits. MY has non-functional mutations in *PSY1* and *CCS. C. chinense* ‘Habanero White’ and ‘White Moruga’ and *C. annuum* ‘Chupetino White’ were purchased from Mojo Pepper (Italy) and grown in the greenhouse of Seoul National University (Seoul, South Korea). All three cultivars have white mature fruit color.

### Nucleic Acid Extraction

Genomic DNA (gDNA) was extracted using a modified cetyltrimethylammonium bromide (CTAB) method (Lee et al., 2017). Leaf tissues were homogenized using 3-mm steel beads with the aid of TissueLyserII (Qiagen, Hilden, Germany). The concentration and purity of gDNA were measured with a Nanodrop spectrophotometer (BioTek, Winooski, VT), and the DNA was then diluted to a final concentration of 20 ng/μL in distilled water for further experiments. Total RNA was also extracted from various tissues. For AC08-045 and AC08-201, RNA was extracted from roots, stems, leaves, and three stages of fruit. RNA was also extracted from immature fruits of the virus-inoculated MY used for VIGS analysis, and from young leaves of *C. chinense ‘*Habanero White’ and ‘White Moruga’ *C. annuum ‘*Chupetino White.’ Total RNA was extracted using MG RNAzol kit (MGmed, Seoul, South Korea) according to the manufacturer’s instructions. Complementary DNA (cDNA) was synthesized from 2 μg of RNA using the EasyScript Reverse Transcriptase kit (TransGen, Beijing, China) with oligo (dT) primers. The resulting cDNAs were used for further analyses.

### PCR Amplification and Sequencing

PCR was performed in a total volume of 25 μL using PrimeStar GXL DNA polymerase (Takara Bio, Kusatsu, Japan) with 100 ng of each gDNA template and 0.5 μL of 10 pmol gene-specific primers for the carotenogenic genes *PSY1*, *PSY2*, *Lcyb*, *CrtZ-2*, *ZEP*, and *CCS*. Five of these genes were amplified using one primer set to amplify the whole gene, while three sets of primers were used for *ZEP* amplification because of its large size: a gDNA length of 4,802 bp and a cDNA length of 1,986 bp. Primers for *ZEP* were designed to cover all exons of the gene and to overlap each amplicon. For *GLK2* and *PRR2*, cDNAs from immature fruits were used as amplification templates, as both genes contain long introns (Table 1). For reverse-transcription PCR (RT-PCR), 2 μL of 4 × -diluted cDNA was used as a template using primers identical to those used for gDNA PCR with following reaction mixture: 0.3 μL EX Taq (Takara, Japan), 2.5 μL 10 × PCR buffer, 2 μL 2.5 mM dNTPs, 0.5 μL 10 pmol primers, and 17.2 μL triple distilled water. The RT-PCR conditions were 28 cycles of 95°C for 30 s, 58°C for 30 s, and 72°C for 90 s. The resulting amplicons were separated on a 1% agarose gel and DNA was recovered using a LaboPass PCR clean-up kit (Cosmo Genetech, Seoul, South Korea). Elution products were either directly sequenced or cloned into a modified T-blunt vector (SolGent, Daejeon, South Korea) prior to sequencing. Plasmid DNA was extracted using an AccuPrep plasmid mini extraction kit (Bioneer, Daejeon, South Korea). Sanger sequencing was performed at Macrogen (Seoul, South Korea) and the nucleotide sequences were analyzed with Lasergene’s SeqMan program (DNASTAR, Madison, WI).

**TABLE 1:** List of primers used to amplify fruit-color-related genes.

**Gene**	**Gene no.**	**gDNA (bp)**	**CDS (bp)**	**Primer sequence (5′ to 3′)**
*PSY1*	*CA04g04080*	2,844	1,260	F: ATGTCTGTTGCCTTGTTATGG R: ATCCTGATTTCATGTTCTTGTAGAAG
*PSY2*	*CA02g20350*	2,985	1,299	F: ATGTCTGTTGCTTTGTTGTGG R: CAACTTCATTCATGTCTTTGTTAGTG
*Lcyb*	*CA05g00080*	1,497	1,497	F: ATGGATACGCTCTTGAGAACC R: TCATTCTTTATCCTGTAACAAATTG
*CrtZ-2*	*CA03g25820*	2,025	948	F: ATGGCTGCTGAAATTTCAAT R: CTTTGATCATAATCTCTTCGAAC
*ZEP* (fragment 1)	*CA02g10990*	1,760	-	F: TCCTTTCACTTCCTTTGGCCT R: AGCTTCACTGTGTCCGAACA
*ZEP* (fragment 2)		1,952	-	F: GAATGGACAACGGTTTACAGGT R: CAAACCACAGGATATCAACTTCC
*ZEP* (fragment 3)		1,709	-	F: GGACTTGGGAATGCCTCTAATG R: ATGCTGTACAAATTTCCCGTTT
*CCS*	*CA06g22860*	1,497	1,497	F: ATGGAAACCCTTCTAAAGCCT R: TCAAAGGCTCTCTATTGCTAG
*GLK2*	*CA10g02900*	5,859	942	F: ATGATGCTTGTTGTATCTACACCA R: GAGGTATTTTTGTAATCCCTTGAC
*PRR2*	*Capana01g000809*	5,588	1,764	F: ATGATTTGCATTGAGGATGAA R: TCATCTCCAACATCGAGAGC

### Carotenoid Extraction and Saponification of AC08-045 and AC08-201

Mature fruits were harvested and the pericarp tissue was sectioned and collected without placenta for carotenoid extraction. Three biological replicates of fruits for each AC08-045 and AC08-201 were used. The carotenoid pigments were extracted according to method of Collera-Zúñiga et al. (2005) but using different volumes of chemicals. Acetone (20 mL) was added to 1 g of freeze-dried pericarp and incubated at 4°C for 20 h to extract the pigments. The extracts were dried by evaporation and incubated with 3 mL acetone, 3 mL methanol, and 1 mL 30% potassium hydroxide/methanol at room temperature for 2 h 30 min in dark condition to avoid carotenoid degradation. After saponification, the extracts were transferred to a separatory funnel with 20 mL diethyl ether, shaken, and left to settle. The extracts were combined with 2 mL of 10% NaCl to separate the phases and to transfer the pigments to the ether. Subsequently, 2 mL of 2% Na_2_SO_4_ was added to remove all water in the ether phase. The ether phase was collected and dried using vacuum concentrator. The resulting dried residue was dissolved in 2 mL acetone and filtered through an Acrodisc syringe filter (13 mm, 0.2 μm) (Pall Corporation, New York, NY).

### Carotenoid Analysis by Ultra-Performance Liquid Chromatography

Carotenoids extracted from mature fruit pericarps of the two AC08 accessions were analyzed using an Acquity UPLC-H-Class system (Waters, Milford, MA). Separation was performed using an Acquity UPLC HSS T3 column (2.1 × 100, 1.8 μm) at 35°C. The mobile phase was a binary solvent system consisting of phase A (acetonitrile/methanol/methylene chloride, 65/25/10, v/v/v) and phase B (distilled water). The gradients were programmed as previously described (Kim et al., 2016). The UV wavelength was set to 450 nm. For qualitative and quantitative analysis of carotenoids, 11 standards were purchased from Sigma-Aldrich (St. Louis, MO): antheraxanthin, capsanthin, capsorubin, lutein, neoxanthin, violaxanthin, zeaxanthin, α-carotene, α-cryptoxanthin, β-carotene, and β-cryptoxanthin.

### High-Resolution Melting Analysis

A pair of primers was designed to amplify the short genomic region (<300 bp) centered on the target *PRR2* target SNP. Genotyping analysis was performed using a Rotor-Gene 6000 real-time PCR (Qiagen, Hilden, Germany). The reaction mixture was prepared in a total volume of 20 μL containing 80 ng of DNA, 0.3 μL of R Taq (Takara Bio), 2 μL of 10 × PCR buffer, 2 μL of 2.5 mM dNTPs, 0.6 μL of SYTO 9 (Thermo Fisher Scientific, Waltham, MA), and 0.5 μL of 10 pmol primers. Sequences of forward and reverse primers used in the study were as following: 5′-TTTGAAAGAGGAGAATGGTTCA-3′ (forward) and 5′-TGAGCTATGGGGACCAGAAG-3′ (reverse). The PCR conditions consisted of 95°C for 5 min; 55 cycles of 95°C for 20 s, 55°C for 20 s, and 72°C for 30 s; and 60°C for 1 min. For high-resolution melting (HRM) analysis, the temperature was increased 0.1°C per every minute from 65°C to 90°C. Melting curve and HRM-normalized graphs were analyzed using Rotor-Gene Q series software 2.1.0.

### Transmission Election Microscopy (TEM)

TEM analysis was conducted using immature and mature fruits of AC08-045 and AC08-201. Both immature and mature fruit pericarps of AC08 accessions were longitudinally sliced to a thickness of 1 mm. Specimen preparation for TEM was done following the method described by Seo et al. (2014). Polymerized samples were than trimmed, sliced to a thickness of 500 nm, stained with toluene blue, and examined under a light microscope. After confirming plastid existence and localization, regions with plastids were sliced to a thickness of 80 nm and observed using a JEM-1400 Flash (JEOL, Tokyo, Japan) (120 kV) transmission electron microscope. Processes from trimming to TEM were kindly done by the Dental Research Institute (Seoul National University, Seoul).

### Virus-Induced Gene Silencing of *PRR2*

Ligation-independent cloning (LIC) for pTRV2-PRR2 construction was conducted as described by Kim et al. (2017). The partial coding sequence of *PRR2* was amplified with the following primers containing 15-bp adapter sequences (underlined): 5′-CGACGACAAGACCCTTAAGTCC TCCTGGGCAACAA-3′ (forward) and 5′- GAGGAGAA GAGCCCTGGTTCAGCAGAATGACTAATGC-3′ (reverse). Purified PCR product was treated with T4 DNA polymerase (Enzymatics, Beverly, MA) along with 5 × blue buffer and 10 mM dATP. *Pst*I digested pTRV2-LIC vector was also treated with T4 DNA polymerase and 5 × blue buffer with 10 mM dTTP. Both reaction mixtures were incubated at 22°C for 30 min followed by 75°C for 20 min. A total of 30 ng of PCR product and 400 ng of TRV2-LIC vector were mixed and incubated at room temperature for 15 min. The mixture was transformed into *Escherichia coli* DH5α competent cells (TransGen). Plasmids were extracted from transformants and sequenced by Macrogen. Plasmids with intact sequences were introduced into *Agrobacterium tumefaciens* strain GV3101 through electroporation at 2.0 kV. For a negative control for VIGS, the construct pTRV2-GFP was used. The pTRV2-LIC and pTRV2-GFP constructs were kindly provided by Dr. Doil Choi at Seoul National University.

*Agrobacterium* carrying pTRV1, pTRV2-GFP, and pTRV2-PRR2 were grown overnight at 30°C in 5 mL LB medium containing rifampicin (50 μg/mL) and kanamycin (50 μg/mL). 4 mL of *Agrobacterium* cultures was harvested by centrifugation and resuspended in 10 mM MES, 10 mM MgCl_2_, and 200 μM acetosyringone solution to a final OD_600_ of 0.7. Cell suspensions were incubated in a rocking incubator at room temperature for 3 h. Cell cultures containing pTRV1 and each pTRV2 construct were mixed at a 1:1 ratio and infiltrated into the abaxial side of both cotyledons of MY seedlings. Inoculated plants were incubated at 16°C in the dark for 1 day and then grown at 25°C with a 16/8-h light/dark photoperiod.

### Expression Analysis of *PRR2* in VIGS-Treated Fruit Using RT-PCR

Mature fruits were harvested from pTRV2-GFP- or pTRV2-PRR2-inoculated MY plants four months after inoculation. Segments of the mature pericarp displaying lighter colors were excised and their RNA was extracted. RT-PCR was conducted to verify the gene silencing using the same reaction mixture and primers to the method above for PCR and sequencing. The RT-PCR conditions were 28 cycles of 95°C for 30 s, 58°C for 30 s, and 72°C for 90 s. The *Actin* was used for internal control with following primers: 5’-ATTCTCACCTTGAAGTATCCCA-3’ (forward) and 5’-ATAGCAACATACATGGCAGG-3’ (reverse). Three biological replicates were used for reproducibility.

### Carotenoid Extraction and Saponification of VIGS-Treated Fruits

Carotenoids were extracted as described by Yoo et al. (2017), with some modifications. The samples were put on ice, and the whole procedure was done under dim light to prevent carotenoid oxidation and degradation. The lighter-colored pTRV2-PRR2-inoculated pericarps and the pTRV2-GFP-inoculated pericarps were diced, immediately frozen in liquid nitrogen, and freeze-dried using a vacuum freezer.

The freeze-dried tissues were ground into fine powder, and about 50 mg of tissue powder was aliquoted into a 2-mL tube with two glass beads. The upper phases of saponified samples were collected and concentrated using a vacuum concentrator until they were fully dried. The dried samples were dissolved with 500 μL HPLC-grade acetone (Honeywell) with sonication, filtered using a 0.2-μm syringe filter (Acrodisc LC 13 mm syringe filter, PVDF membrane; Pall, NY, United States), and bottled in a 1.5-mL HPLC amber vial to prevent light exposure. HPLC analysis of carotenoids was performed by the NICEM chromatography lab (Seoul National University). Seven carotenoids were used as standards: capsanthin, capsorubin, lutein, zeaxanthin, α-carotene, β-carotene, and β-cryptoxanthin.

## Results

### The White-Fruited Accession AC08-201 Accumulates Minimal Levels of Carotenoids

**FIGURE 1:**
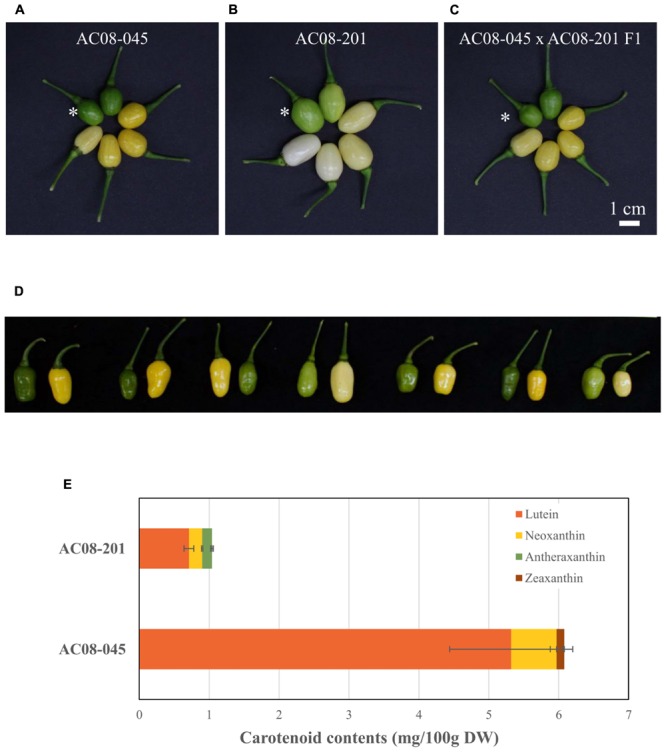
Pepper fruits and carotenoid profiles of AC08-201 and AC08-201 showed less carotenoid accumulation in AC08-201 fruit. **(A–C)** Pepper fruits are displayed in a clockwise di ection according to the ripening stages. An asterisk indicates fully expanded immature fruits. **(A)** In AC08-045, fruit color changes from dark green to yellow and then light yellow. **(B)** In AC08-201, fruit color starts out light green and becomes light yellow to white. **(C)** Fruit color of an F_1_ hybrid is similar to that of AC08-045. Scale bar, 1 cm. **(D)** F_2_ individuals from cross between AC08-045 and AC08-201. Seven pairs of immature and mature fruit from each F_2_ individuals were selected from F_2_ population and shown in the figure. **(E)** Carotenoid profiles of AC08-201 and AC08-201. Carotenoids extracted from mature fruit pericarp. Levels of four major carotenoids, lutein, neoxanthin, antheraxanthin, and zeaxanthin, are shown in the graph. Horizontal bars indicate standard deviations of each carotenoid content.

*Capsicum frutescens* AC08-045 bears dark green immature fruit, which change to yellow and then light yellow as they ripe (Figure 1A). AC08-201 fruits start out light green and change to off-white and finally white at maturity (Figure 1B). F_1_ hybrids obtained from a cross between AC08-045 and AC08-201 showed dominance of light yellow over white (Figure 1C); consistently with this, the F_2_ population showed a roughly 3:1 ratio of dark green/light yellow to light green/white.

To investigate the differences in carotenoid composition and levels between the two accessions, we analyzed the carotenoid contents of their mature fruits by UPLC. Among 11 carotenoids, only three were detected in each accession; lutein and neoxanthin in both accessions, zeaxanthin only in AC08-045 and antheraxanthin only in AC08-201 (Figure 1E and Supplementary Table S1). Lutein was the major carotenoid in both accessions. In AC08-045, it accounted for 88% of total carotenoid content, followed by neoxanthin and zeaxanthin. In AC08-201, lutein accounted for 70% of total carotenoids, followed by neoxanthin and antheraxathin. The main differences between two accessions were total carotenoids content and the ratio of zeaxanthin and antheraxanthin in each accession: the total carotenoid content was six times higher in AC08-045 (6.07 ± 0.97 mg/100 g DW) than in AC08-201 (1.02 ± 0.08 mg/100 g DW). Although the colors of zeaxanthin and antheraxanthin are slightly different, these two pigments were least in each accession. These results suggest that the color difference between the two accessions is due to their abundance rather than composition of carotenoids.

### AC08-201 Has Mutations in *CCS*, *GLK2*, *PRR2*, and *PSY1*

To identify the gene(s) controlling the carotenoid levels in AC08-201, we amplified fruit-color-related genes and sequenced the resulting amplicons. We analyzed six carotenoid biosynthetic genes, *PSY1*, *PSY2*, *Lcyb*, *CrtZ-2*, *ZEP*, and *CCS*, along with two genes regulating plastid compartment size, *GLK2* and *PRR2.* Amplicons of the genes except *GLK2* showed the sizes expected based on the reference genome (CM334 v1.55), and no size differences between the two accessions were observed in any of the candidate genes, although for *GLK2*, the amplicons of both accessions were slightly smaller than expected based on the reference genome (Supplementary Figure S1).

**FIGURE 2:**
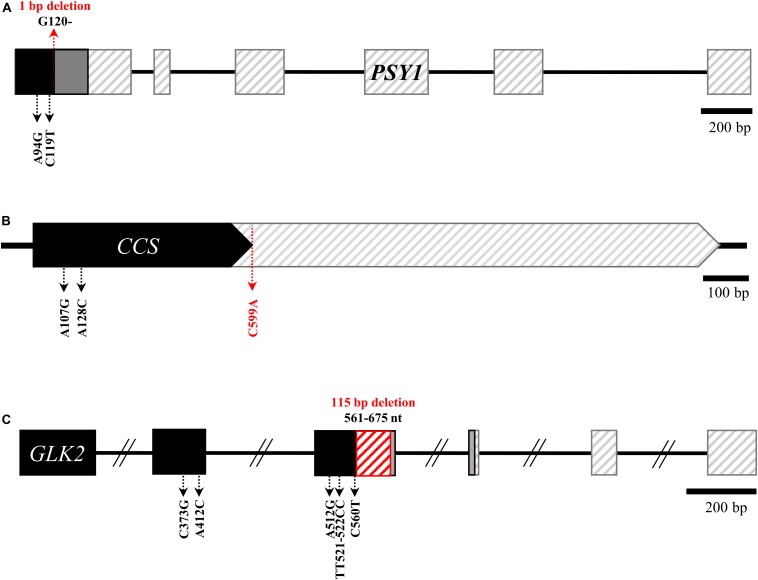
Common background mutations in *PSY1*, *CCS*, and *GLK2* of AC08-045 and AC08-201. **(A–C)** Common mutations in the genes *PSY1*, *CCS*, and *GLK2* of AC08. In alleles with early stop codons, non-synonymous mutations downstream from those stop codons are omitted. Major mutation of each gene are highlighted in red letters. Black region indicates normal transcription, and gray regions indicate frameshifted regions. Regions after early translation termination are indicated with diagonal lines. The 115-bp deletion in *GLK2* is indicated with red diagonal lines.

**FIGURE 3:**
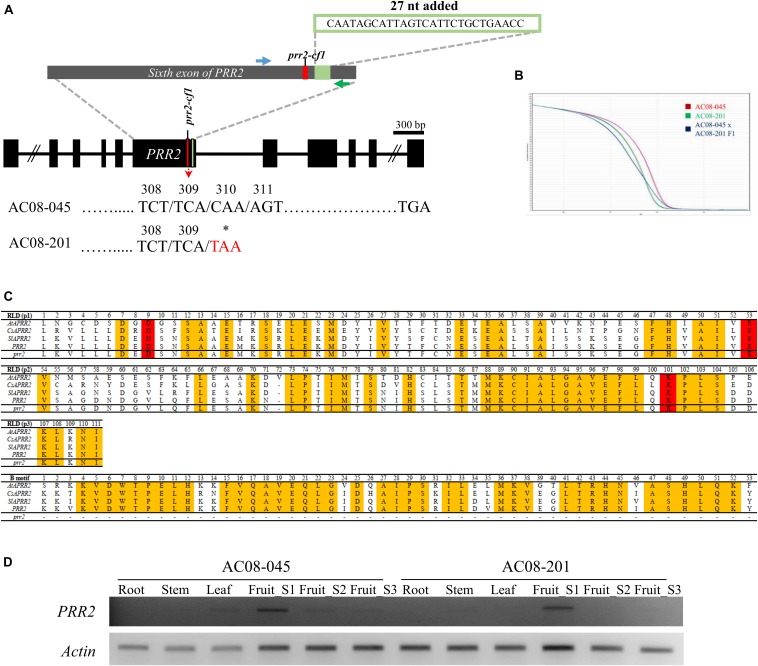
Mutation and HRM markers of *PRR2* in AC08-045 and AC08-201, alignment of *PRR2* domain between Arabidopsis and three horticultural crops, and expression analysis of *PRR2*. **(A)** Comparison of *PRR2* variation between AC08-045 and AC08-201. Variation in the nucleotide sequence and resulting codon change are highlighted in red. A premature stop codon due to a 1-bp substitution was detected in AC08-201. The sixth exon of *PRR2* is expanded in the gray exon. Nucleotide sequence of 27-nt insertion observed in this study compared to Zunla v2.0 (*Capana01g000809*) is shown above. The positions of the designed HRM marker are shown in blue (forward primer) and green (reverse primer). Asterisks indicate stop codons. **(B)** HRM genotyping of *PRR2*. Melting curves are differentiated according to the accessions. Red, green, and blue lines indicate maternal, paternal, and heterozygous type of *PRR2* genotype. *x* axis shows temperature and *y* axis shows normalized fluorescence. **(C)** Alignment of *PRR2* domain between Arabidopsis and three horticultural crops. *AtAPRR2*, *CsAPRR2*, *SlAPRR2*, and *PRR2* refer to the *PRR2* genes of *Arabidopsis thaliana*, *Cucumis sativus*, *Solanum lycopersicum*, and *Capsicum frutescens*, respectively. RLD for receiver-like domain; B-motif for DNA-binding domain. **(D)** Expression pattern of *PRR2* in various tissues. Results of RT-PCR amplification of *PRR2*. Roots, stems, leaves, and three stages of fruits were analyzed. In both AC08-045 and AC08-201, *PRR2* was exclusively expressed in immature stage (Fruit_S1). Actin served as a control. S stands for stage.

Sequence analyses revealed no mutations causing amino acid changes in *PSY2*, *Lcyb*, *CrtZ-2*, or *ZEP.* By contrast, we identified the same structural mutations of *GLK2*, *PSY1*, and *CCS* in both accessions, and a mutation in *PRR2* in AC08-201 only. The three mutations in both accessions were a frameshift mutation due to a 1-bp deletion at nucleotide (nt) position 120 in *PSY1* (Figure 2A); a nonsense mutation (serine to stop codon) in *CCS* due to a cytosine-to-adenine substitution at nt 599 (Figure 2B); and a 115-bp deletion in *GLK2* from nt 561 to nt 675, which causes premature translational termination (Figure 2C). The alignment of the *PSY1*, *CCS*, and *GLK2* coding sequences between the AC08 accessions and CM334 is shown in Supplementary Material S1. The *PRR2* mutation in AC08-201 was a nonsense mutation (cytosine to thymine) at nt 928 (Figure 3A). We designed a HRM marker to discriminate this mutation from the wild-type allele of AC08-045 and used this in our further studies (Figures 3A,B).

We compared the truncated protein sequences of AC08-201 to previously reported sequences from species including Arabidopsis, cucumber (*Cucumis sativus*), and tomato (Figure 3C). Sequence alignment analysis revealed that the deleted region is crucial for the protein’s enzymatic activity, as it contains a DNA-binding motif conserved in transcription factors and a functional domain for nuclear localization (Jiao et al., 2017).

### White Color in Peppers Is Controlled by a Single Recessive Gene

To test whether the nonsense mutation in *PRR2* resulted in the lighter fruit color in AC08-201, we prepared a population segregating for white fruit color by crossing AC08-045 and AC08-201. All fruits from the F_1_ progeny showed a light yellow color indicating that light yellow is dominant over white color. In the F_2_ population, light yellow and white fruits were observed in a ratio of 170:57, which fit a 3:1 ratio with an χ^2^ value of 0.001468 (*P*-value 0.9694) (Table 2). During phenotyping, we observed an interesting trend between immature fruit color and mature fruit color. Fruits with a dark green immature color became light yellow when ripe, whereas fruits with light green immature color turn white when fully mature. This trend of immature fruits with more intense color (dark green) becoming more dark mature fruits (light yellow) indicates that the white fruit-color phenotype is related to plastid development. To test the co-segregation of the mature fruit phenotype and the *PRR2* genotype, we used the HRM marker targeting the nonsense mutation (C928T in cDNA, C2571T in gDNA) (Figure 3B). The genotypes co-segregated completely with the colors, which makes *PRR2* a strong candidate gene for controlling the white fruit color in AC08-201.

**TABLE 2:** Segregation of phenotypes in a F_2_ population.

**Population**	**Size**	**Phenotype**		**Expected ratio**	***p*-value**
		**Dark green then light yellow**	**Light green then white**		
				
AC08-045 × AC08-201 F_2_	227	170	57	3:1	0.97

### *PRR2* Is Expressed in Immature Fruit Pericarps

To evaluate the expression of *PRR2*, we performed RT-PCR using fruit pericarps harvested at different ripening stages, including the immature (S1), breaker (S2), and mature stage (S3). We also analyzed the expression of *PRR2* in root, stem, and leaf tissues (Figure 3D). In both accessions, we observed transcripts of the expected size predominantly at the immature stage (S1), whereas they were almost undetectable in more mature fruits (stages S2 to S3) and in the vegetative tissues. The relative transcript levels of *PRR2* were slightly less than that of actin, which was used as a control. This result demonstrates the expression pattern of *PRR2*, which expressed specifically in immature fruit.

### PRR2 Regulates Plastid Development in Pepper Fruits

**FIGURE 4:**
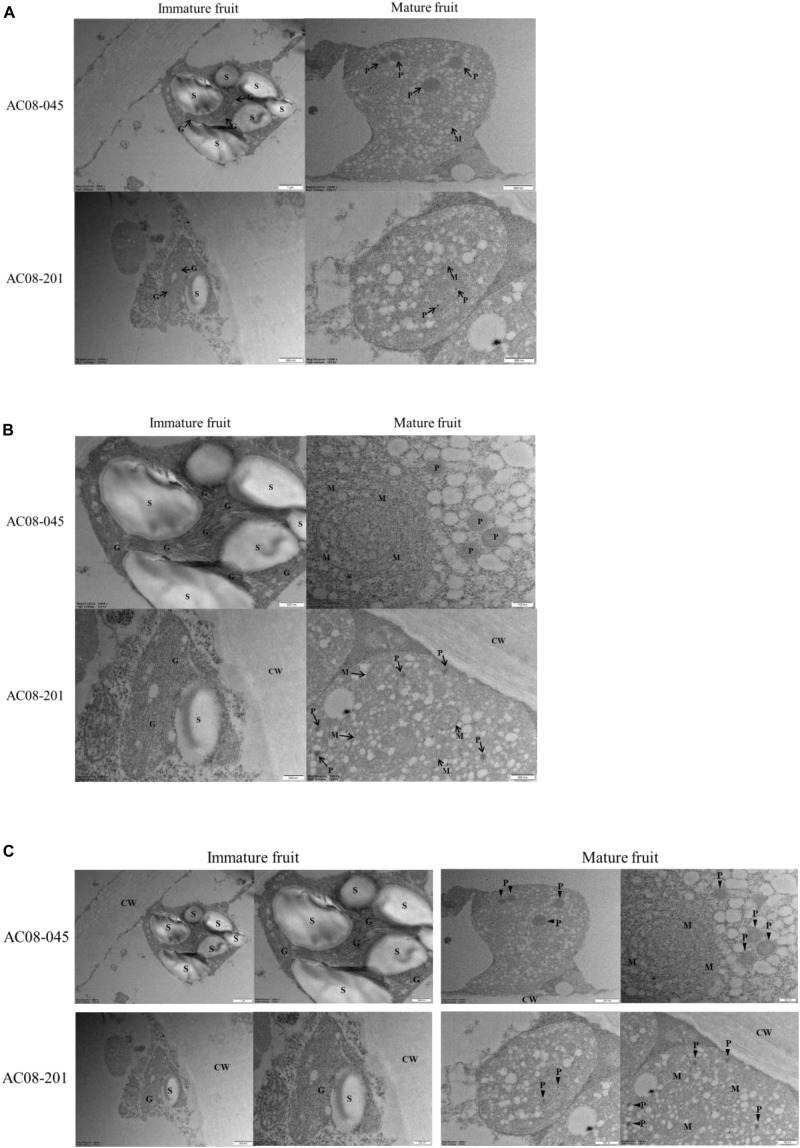
Plastid structures of AC08-045 and AC08-201 observed by TEM. **(A–C)** Chloroplast of immature fruits and chromoplast of mature fruits were observed. **(A)** Images of single plastids of immature and mature fruits of AC08-045 and AC08-201, shown in wide field. **(B)** Magnified images showing inner structures of each plastid. **(C)** Combined image of A and B for comparison. S, starch grain; CW, cell wall; G, granum; P, plastoglobule; M, internal membrane structure. Scale bars for each micrograph are located at the bottom right.

To study how *PRR2* control fruit color, we conducted TEM analysis of fully expanded immature and mature fruits of AC08-045 and AC08-201, using a light microscope and toluene blue-stained samples. We observed a much smaller number of plastid-like structures in AC08-201 than in AC08-045 in both mature and immature fruits. All the cell organelles were displaced toward the cell wall, possibly due to the large tonoplast that develops in the fruit pericarp during fruit maturation (Supplementary Figure S2). We also observed the inner structures of the plastids using TEM. A single plastid from each sample is shown in Figure 4A for size comparison. Chloroplasts were observed in immature fruits of both accessions, with the characteristic thylakoid membrane structure and starch grains. AC08-201 had much smaller chloroplasts than AC08-045, almost half the size, as indicated by the scale bar. The starch grains in the chloroplasts were also larger and more numerous in AC08-045 than in AC08-201. Chromoplasts, which we observed in mature fruit pericarp samples, showed no significant size difference between the accessions. Enlarged images of the inner structures of each plastid are shown in Figure 4B. In immature fruits, chloroplasts in AC08-045 had much thicker thylakoids than those of AC08-201, which were almost undetectable even in magnified images. Moreover, the thylakoids in AC08-045 were highly stacked into well-developed granum, in contrast to those of AC08-201. The plastoglobuli in the chromoplasts were much smaller in mature AC08-201 fruits compared than mature AC08-045 fruits, although the number of plastoglobuli did not differ significantly. The plastoglobule is the inner structure of the plastid, which mainly accumulates carotenoids and contains the carotenoid biosynthetic enzymes of chromoplasts; thus, the number and size of plastoglobuli are closely related to the chromoplast carotenoid content (Oleszkiewicz et al., 2018). Images of a single plastid and its inner structures are collected in Figure 4C to provide a comprehensive comparison.

### Silencing of *PRR2* Leads to Lighter Yellow Fruit Color in MY

**FIGURE 5:**
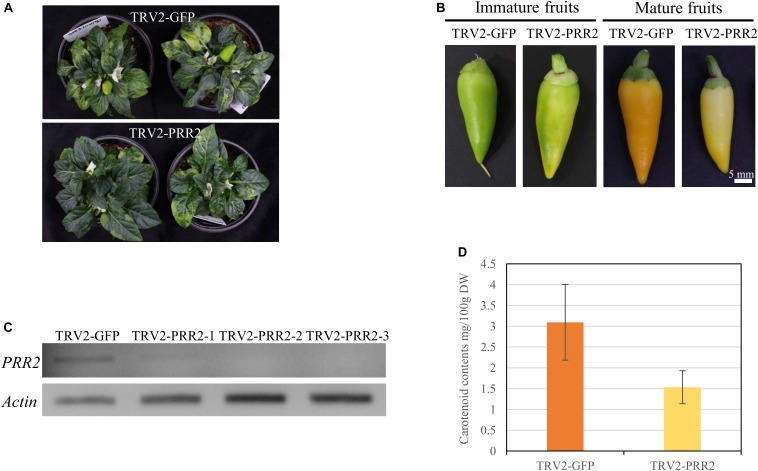
VIGS of *PRR2* in MY. **(A)** Phenotypes of TRV2-GFP-inoculated MY (top) and TRV2-PRR2-inoculated MY (bottom) plants at 60 days after inoculation (DAI). **(B)** Effect of the VIGS of *GFP* and *PRR2* on the color of immature and mature fruit. Scale bar, 5 mm. **(C)** Expression analysis of *PRR2* using RT-PCR. Three biological replicates of *PRR2*-silenced fruits were used. *Actin* served as a control. **(D)** Total carotenoid contents of TRV2-GFP- or TRV2-PRR2-inoculated MY fruits. The standard deviation of carotenoid contents of TRV2-GFP- or TRV2-PRR2-inoculated MY fruits were 0.909484 and 0.396838, respectively, with the *p-*value of 0.09078 using Student’s *t*-test. TRV2-GFP was used as a vector control.

We evaluated the function of *PRR2* using *C. annuum* ‘MY,’ which has non-functional mutations in both *PSY1* and *CCS*, via TRV-mediated VIGS. *Agrobacterium* carrying pTRV2-GFP served as a negative control. Neither TRV2-GFP- nor TRV2-PRR2-inoculated plants displayed any significant differences from the un-inoculated wild-type MY plants, except for the slight discoloration due to the TRV symptoms (Figure 5A). The flowers and fruits of silenced plants developed at about 60 and 75 DAI, respectively. There were no visible changes in the flower colors in either group, nor in the colors of the leaves (data not shown). In the pTRV2-GFP-inoculated plants, the immature fruits were dark green in color, whereas the immature fruits in the *PRR2-*silenced plants showed patches of ivory to pale green color. The mature fruit color showed a similar trend: the pTRV2-GFP-inoculated fruits were yellow, whereas the *PRR2-*silenced fruits were ivory to light yellow (Figure 5B). The fruit-specific effect of TRV2-PRR2 inoculation support our hypothesis that *PRR2* is *C1*, as *C1* does not affect the vegetative tissue phenotype.

We conducted RT-PCR to test the expression levels of *PRR2* in the pTRV2-GFP-inoculated and *PRR2*-silenced plants. The cDNA of immature fruits was used for RT-PCR, as *PRR2* is predominantly expressed in immature fruits and barely expressed in mature fruits (Figure 3D). Whereas the fruits of the pTRV2-GFP-inoculated plants showed *PRR2* expression, there was almost no detectable *PRR2* expression in the fruits from the *PRR2-*silenced plants, indicating the successful post-transcriptional gene silencing (PTGS) of the target gene (Figure 5C).

We used HPLC analysis to obtain a quantitative and qualitative measurement of the carotenoid contents of the mature fruits from the TRV-GFP- and TRV2-PRR2-inoculated plants. The total carotenoid contents were 3.09 mg/100 g DW and 1.54 mg/100 g DW, respectively, with non-significant *P*-value according to Student’s *t*-test. The mature fruits of *PRR2*-silenced plants had less than half the carotenoid content of those from the control plants. Lutein was the main component in fruits from both the TRV2-GFP- and TRV2-PRR2-inoculated plants, and α-carotene and β-carotene were also detected in the former (Figure 5D and Supplementary Figure S3).

### Several White Pepper Accessions Carry *PRR2* Mutations

To validate *PRR2* mutations are commonly observed in other *Capsicum* spp., we analyzed the cDNA sequences of *PRR2*, along with *PSY1* and *CCS*, in three other white pepper accessions: *C. chinense* ‘Habanero White’ and ‘White Moruga’ and *C. annuum* ‘Chupetino White.’ Non-functional alleles of *PSY1*, *CCS*, and *PRR2* were present in all three accessions, although the types of mutation were slightly different between accessions.

**FIGURE 6:**
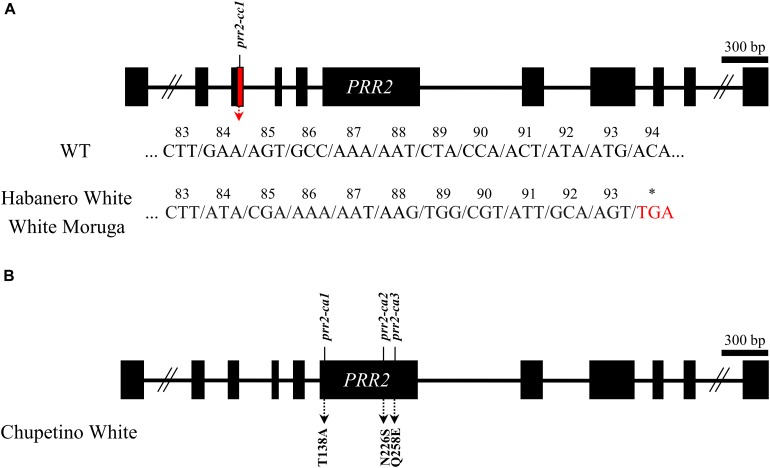
Structural mutations of *PRR2* in pepper cultivars with white mature fruit. **(A)** *C. chinense* ‘Habanero White’ and ‘White Moruga’ had same structural mutation leading to a premature stop codon. Consecutive non-synonymous mutations are shown in the figure. **(B)** *C. annuum* ‘Chupetino White’ had three non-synonymous substitutions. The amino acid substitutions are shown in the figure.

In *PSY1*, Habanero White and Chupetino White had an early stop codon in the cDNA sequence at 679 nt, along with some non-synonymous mutations (N16S and A40V in Chupetino White; A40V in Habanero White). White Moruga had a 28-nt deletion in the coding sequence at nt 1,103, which also leads to early translation termination of the *PSY1* gene, along with one non-synonymous mutation resulting in a G62R substitution in the amino acid sequence. In *CCS*, all three accessions have several nucleotide substitutions (resulting in the amino acid alterations K36R and Y43S), including the common SNP leading to an early stop codon at nt 599 (data not shown). Along with the early stop codons of *PSY1* and *CCS*, these accessions also had structural mutations in *PRR2*. Habanero White and White Moruga had the same structural mutation of *PRR2*, which results in an early stop codon at amino acid 93 of the protein (Figure 6A). Chupetino White had three non-synonymous mutations in the sixth exon of *PRR2* (Figure 6B). Taken together, these results indicate that pepper accessions with white mature fruits commonly carry mutations in *PSY1*, *CCS*, and *PRR2*. Taken together, our results suggest that *PRR2* might be the gene corresponding to *C1*, the last unidentified locus governing pepper mature fruit color.

## Discussion

In *Capsicum*, a three-locus model (*C1*, *C2*, and *Y*) has been proposed to explain fruit-color inheritance (Hurtado-Hernandez and Smith, 1985). The genes at *C2* and *Y* were previously reported to encode phytoene synthase and capsanthin-capsorubin synthase, respectively. In this study, we verified that the *C1* locus contains the gene *pseudo response regulator2-like* (*PRR2*), and the white color of AC08-201 is due to triple mutations in *PSY1* (*C2*), CCS (*Y*), and *PRR2* (C1). According to this hypothesis, the fruits of plants with a functional *C1* allele are expected to be more intensely colored than those of plants with a non-functional *C1* allele: red (*C1*, *PSY1*, *CCS*) vs. light red (*c1*, *PSY1*, *CCS*); orange (*C1*, *psy1*, *CCS*) vs. pale orange (*c1*, *psy1*, *CCS*); orange-yellow (*C1*, *PSY1*, *ccs)* vs. lemon-yellow (*c1*, *PSY1*, *ccs*); pale orange-yellow (*C1*, *psy1*, *ccs*) vs. white (*c1*, *psy1*, *ccs*). As the *C1* genotype does not affect the overall color category (red, orange, yellow, or white), *C1* was proposed to be a regulatory gene of carotenoid biosynthesis rather than a structural gene. In accordance with this hypothesis, our results strongly suggest that *C1* corresponds to *PRR2*.

In the previous study, Pan et al. (2013) showed that *APRR2* overexpression in tomato largely enhanced the levels of chlorophyll in immature fruits and slightly enhanced the levels of carotenoids in red fruits by increasing plastid number and area. The same group also reported a premature stop codon due to a guanine-to-adenine substitution at 1,404 nt in *PRR2* (based on the coding sequence of *Capana01g000809*) in sweet peppers with white immature fruits. In cucumber, *PRR2* is reported to control the green immature fruit color, and its mutated allele, *prr2*, causes a white color. *prr2* encodes a truncated 101-amino-acid protein due to a frameshift mutation that creates a premature stop codon (Liu et al., 2016). All previous studies of the function of *PRR2* have focused mainly on the immature stage of the fruits. However, in this study, we showed that *PRR2* affects the color of mature fruits. Given that chromoplasts, the differentiated form of proplastids that are the primary sites of carotenoid accumulation, derive from chloroplasts (Lu et al., 2006; Jarvis and López-Juez, 2013), a larger chloroplast compartment size at the immature stage could result in a larger chromoplast compartment at the mature stage, which could accumulate more carotenoids. However, a contrasting report indicates that increased chlorophyll content is not always statistically linked to increased carotenoid content in peppers (Brand et al., 2012), and thus more studies will be required to clarify the function of *PRR2*.

During sequencing analysis, we observed that the sequences of *PRR2* in the accessions we sequenced are different from that of the reference genome. Pan et al. (2013) reported that the coding sequence of *PRR2* consisted of 1,683 bp (GenBank accession no. KC175445). In this study, however, we performed RT-PCR using primers that cover the full length of the gene and identified bigger amplicons (total length 1,764 bp). The resulting sequences were very similar to the sequence of this gene derived from the *C. annuum* Zunla cultivar genome, v2.0 (*Capana01g000809*), which is differed by 54 bp in sixth exon of the gene compared to KC175445 sequence, although the resulting sequence also differed in length, by 27 bp (total length 1,737 bp) compared to the Zunla reference. For verification, we sequenced *PRR2* from other accessions, including *C. annuum*. The sequencing results showed the same extra 27 bp of sequence, located in the sixth exon of the gene (Figure 3A). The coding sequences of *PRR2* in AC08-045 and AC08-201 are shown in Supplementary Material S2.

We classified the ripening stages of each accession into three stages. In AC08-045, fruit color changes from dark green to yellow and then light yellow. In AC08-20l, fruits become light green, ivory, and then white. However, if *PRR2* is the only candidate for regulating color intensity, fruits of both accessions would be expected to have only two ripening stages. In other words, once the plastid compartment size is determined by *PRR2* and then the chloroplasts transition into chromoplasts during ripening, the fruit color must become yellowish, and the color would be expected to be maintained. It was assumed that the original classification of ripening into more than two stages might have resulted from degradation of carotenoids during ripening. Hurtado-Hernandez and Smith (1985) also reported that some mature pepper fruits lose pigment as they move from physiological maturity to over-maturity. Ha et al. (2007) studied the carotenoid profiles and genotype of *C. annuum* IT158782, with white mature fruit. They observed that *PSY1* and *CCS* could not be amplified, indicating the presence of structural mutations abolishing the functions of those genes. In this context, IT158782 and AC08-201 can both be considered to have non-functional alleles of *PSY1* and *CCS*, although the alleles may be different. In IT158782 fruits, however, no carotenoids were detected, including lutein, the major carotenoid component of AC08-201. This might be due to the difference in harvest time. Indeed, IT158782 fruits accumulated 1.28 μg/g (0.13 mg/100 g DW) of lutein before they were fully mature. If the carotenoid extraction of AC08-201 were performed with more fully ripened fruits, no carotenoids might be detected.

In this study, we observed the *PRR2* gene was mainly expressed in immature fruits indicating that carotenoid accumulation in mature fruit is affected by the early expression of *PRR2*. To understand the discrepancy between *PRR2* expression and carotenoid accumulation, we observed the plastid structures of both immature and mature fruit pericarps of AC08 accessions as the *PRR2* is reported to regulate the plastid number and area. Immature fruits of AC08-201 had smaller chloroplasts and less developed thylakoid structures, which were stacked only one or twice into each granum. Although the chromoplast sizes in mature fruits were not significantly different, the plastoglobuli were larger in AC08-045, with a normal *PRR2* gene. As the plastoglobule, the inner structure of the plastid, is where the carotenoids mainly accumulate, it is plausible that its size could be closely related to carotenoid content in chromoplast (Oleszkiewicz et al., 2018). Considering the fact that conversion from chloroplast to chromoplast is one of the most important developmental changes in fruit ripening, thylakoid structures of chloroplast are dissembled and plastoglobuli are formed in chromoplast (Klee and Giovannoni, 2011), indicating that expression of *PRR2* in immature fruit might be connected to the carotenoid accumulation in mature fruit. This also supports our theory that *PRR2* is a carotenoid-content-regulating gene, as carotenoid accumulation could be limited by the smaller compartment space due to the *PRR2* mutation. However, the mechanism regulating plastid development still needs to be studied.

We also performed VIGS analysis of *PRR2*, using the accession *C. annuum* ‘MicroPep Yellow (MY),’ which has non-functional *PSY1* and *CCS* alleles and a functional *PRR2* gene. Therefore, MY and AC08-045 belong to the same group according to the three-locus model. Silencing of *PRR2* resulted in lighter color of immature and mature fruits in MY plants, similar to those of AC08-201 with non-functional *PRR2*. This result supports our hypothesis about the role of *PRR2* in controlling fruit color in peppers that produce pale orange-yellow and white fruits.

We also analyzed the coding sequences of *PSY1*, *CCS*, and *PRR2* in other pepper accessions with white mature fruit (Habanero White, Chupetino White, and White Moruga) and identified several significant mutations in all three genes. All three cultivars carried non-synonymous mutations and an early stop codon in *PSY1* and *CCS*, respectively. In *PRR2*, Habanero White and White Moruga had an early stop codon, and Chupetino White had three non-synonymous mutations (Figure 6). The portion of PRR2 deleted in Habanero White and White Moruga contained a receiver-like domain (RLD) and DNA-binding domain (B-motif), and the mutations in Chupetino White were mostly located in the part of the gene encoding the golden-2 like transcription factor domain, which is critical for the normal function of APRR2 (Jiao et al., 2017). To sum up, all white accessions used in this study had *prr2/psy1/ccs* genotypes. However, *PRR2* mutations do not appeared to be conserved within species as mutations found in this study were different from those reported by Pan et al. (2013) and Borovsky et al. (2019) in *C. annuum* and *C. chinense*. Furthermore, the mutations in *PSY1* and *CCS* are also not species specific as the various allelic variations were previously reported even in same species (Ha et al., 2007; Jeong et al., 2018). However, to confirm that *PRR2* is the gene corresponding to the *C1* locus, more study is still needed in *PSY1/CCS*, *psy1/CCS*, and *PSY1/ccs* pepper of other background genotypes.

Borovsky et al. (2019) comprehensively demonstrated the effects of *CcLOL1, GLK2*, and *PRR2* on chloroplast biogenesis. In fact, *CcLOL1* is closely located very closely to *PRR2* and has similar function. Therefore, we cannot fully rule out the effect of *LOL1* in mature color development of white pepper accessions. However, the VIGS study using MY supports our hypothesis that the gene determining white mature color in these accessions is *PRR2* as we used *PRR2* specific sequence to induce gene silencing. In the further study, the relationship between *GLK2, LOL1*, and *PRR2* on chromoplast biogenesis needs to be revealed as chromoplast can be converted from chloroplast.

In conclusion, in this study we have demonstrated the function of *PRR2*, the regulator of color intensity in both *PSY1*- and *CCS*-mutated genotypes. Silencing of *PRR2* by VIGS resulted in lighter fruit color. Moreover, although there were some differences in the specific mutations, non-functional *PRR2* alleles are common in white pepper accessions of the three major *Capsicum* species, along with non-functional alleles of *PSY1* and *CCS*. This is the first report revealing the role of *PRR2* in mature fruit color and the identity of the gene at the *C1* locus.

## Data Availability Statement

The raw data supporting the conclusions of this article will be made available by the authors, without undue reservation, to any qualified researcher.

## Author Contributions

H-BJ conducted the VIGS, UPLC, and sequence analysis of genes in the AC08 cultivar, while S-JJ conducted VIGS, microscopy, expression analysis, and sequence analysis of other cultivars. Both wrote the manuscript and made the figures. M-YK supervised VIGS experiment. J-KK won supervised TEM microscopy, and SK kindly lent machine for UPLC for carotenoid analysis. B-CK supervised the overall processes and revised the manuscript.

## Conflict of Interest

The authors declare that the research was conducted in the absence of any commercial or financial relationships that could be construed as a potential conflict of interest.
