# Supplementary material for: Candidate Gene Analysis Reveals That the Fruit Color Locus C1 Corresponds to PRR2 in Pepper (Capsicum frutescens)
Source: Front Plant Sci. 2020 Apr 9;11:399. doi: 10.3389/fpls.2020.00399 (PMC7161348; doi:10.3389/fpls.2020.00399)
Supplement: Supplementary file 2 [file Data_Sheet_2.pdf]

27 bp insertion in coding seq. in grey box

9 aa insertion in amino acid seq. in blue bot

SNP leading to stop codon in red box

Translation termination in yellow box

>AC08-045 *PRR2* coding seq.

ATGATTTGCATTGAGGATGAATTATTGGGTTGGAAAGATTTCCCAAAGGGGCTTAAAGTCCTGCTCCTTGATGAAGATAGCAACTCTGCTGCTGAGATGAAATCAAGGCTTGAG
AAAATGGACTATATAGTCTACACGTTCTGCAATGAGAGTGAAGCTTTGTCTGCAATCTCTAGCAAATCCGAGGGCTTTCATGTTGCCATTGTGGAGGTAAGTGCAGGCGATAATG
ATGGGGTCCTCCAATTTCTTGAAAGTGCCAAAAATCTACCAACTATAATGACATCAAATATTCATTCTCTCAGCACAATGATGAAGTGTATTGCGCTTGGTGCAGTTGAGTTCCTT
CAGAAACCATTATCAGATGACAAACTCAAAAATATATGGCAGCATGTAGTTCACAAGGCATTCAATGCTAGAAAGGATGTGTCCGGACCACTTGAGCCGGTAAAAGAATCTCTT
CTTTCGATGCTACAGCTACAACCAGAAAAGGGTGAACCAGATGACAAAAGTTCAAATGGAACAGAACCTCTCATTGCAGTTGCGGACAACAATACCGAACAGTCATCAGGCT
GTGATAAATACCCTGCTCCCTCAACCCCTCAATTGAAACAAGGAGTGCGGTCAGTGGATGATAGTGACTGCCATGATCATACTATCTTCTCAACTGACCAAGACAGTGGGGAGC
ATGATGGTGACACTAAATCCGTCGAAACTACTTATAACAATTCACTTGCTGAGAATACTGTCCAAATAAGTCCTCCTGGGCAACAAGAAGATATAATTTTGAAAGAGGAGAATG
GTTCATCTCCTCATCAAACTATGGAGGCTGATATTGCTACCTTTTCACAAATTAATGACTGCGCTGACAATAGTGATGGTTCATCTCCTCATCAAAAGACGGAGGCTGATATTGCT
ACTACCTCTTCACAAAGTAAAGACTGCCCTGACAATAGCATTAGTCATTCTGCTGAACCTAGTAAAGCTTCTGGTCCCCATAGCTCAAGTGGGACTAAATCCAATAAGAAAAAG
GTGAAGGTAGATTGGACACCTGAACTACACAAAAAGTTTGTTCAAGCAGTAGAGCAACTTGGTATAGATCAAGCCATTCCTTCTCGAATACTAGACGTGATGAAAGTGGAGGG
CTTAACGAGACATAACATAGCTAGCCATCTCCAGAAATACAGAATGCATCGGAGGCAAATTTTGCCAAGGGAAGTGGAAAGGAGATGGCCCCATCCGCAACCTAGAGATTCA
GTACAAAGGAATTACTATCCTCATAAACCTGTCATGACATTCCCACCATATCATTCTAATCATGTCGCCCCAGCTGGTCAATGTTATCCTGCTTGGGTACCACCGGCTAGTTATCCG
AATGGTTTACAAGTGTGGGGTTCACCTTACTATCCGGGATGGCAGCCTGCAGAGACTTGGCACTGGAAGCCTCATCCAGGGCTGCTTGCTGATACATGGGGCTCCCCTGTCAT
GCCACCATCGTTTGGATCGTATCCACCATATCCCCAGAATGCTGGAATGTACCAGTCTCACGGAATGCATAACAGATATAGCATGCTAGAGAAGTCATTCGATGTTCACCCGGCA
GAGGAGGTGATTGATAAAGTAGTAAAGGAGGCAATAACCAAACCATGGTTACCACTTCCTTTGGGCTTAAAACCTCCTTCCATGGAGGGTGTACTCGACGAGCTTTCTAGACA
AGGAATCTCAACCGTCCCTCCACGAATCAATGGCTCTCGATGTTGGAGATGA

>AC08-201 *PRR2* coding seq.

ATGATTTGCATTGAGGATGAATTATTGGGTTGGAAAGATTTCCCAAAGGGGCTTAAAGTCCTGCTCCTTGATGAAGATAGCAACTCTGCTGCTGAGATGAAATCAAGGCTTGAG

AAAATGGACTATATAGTCTACACGTTCTGCAATGAGAGTGAAGCTTTGTCTGCAATCTCTAGCAAATCCGAGGGCTTTCATGTTGCCATTGTGGAGGTAAGTGCAGGTGATAATG

ATGGTGTCCTCCAATTTCTTGAAAGTGCCAAAAATCTACCAACTATAATGACATCAAATATTCATTCTCTCAGCACAATGATGAAGTGTATTGCGCTTGGTGCAGTTGAGTTCCTTC

AGAAACCATTATCAGATGACAAACTCAAAAATATATGGCAGCATGTAGTTCACAAGGCTTTCAATGCTAGAAAGGATGTGTCCGGACCACTTGAGCCGGTAAAAGAATCTCTTC

TTTCGATGCTACAGCTACAACCAGAAAAGGGTGAACCAGATGACAAAAGTTCAAATGGAACAGAACCTCTCATTGCAGTTGCGGACAACAATACCGAACAGTCATCGGGCTG

TGATAAATACCCTGCTCCCTCAACCCCTCAATTGAAACAAGGAGTGCGGTCAGTGGATGATAGTGACTGCCATGATCATACTATCTTCTCAGCTGACCAAGACAGTGGGGAGCA

TGATGGTGACACTAAATCCGTCGAAACTACTTATAACAATTCACTTGCTGAGAATACTGTCCAAATAAGTCCTCCTGGGCAACAAGAAGATATAATTTTGAAAGAGGAGAATGGT

TCATCTCCTCATCAAACTATGGAGGCTGATATTGCTACCTTTTCACAAATTAATGACTGCGCTGACAATAGTGATGGTTCATCTCCTCATCAAAAGACGGAGGCTGATATTGCTAC

TACCTCTTCA**T**AAAGTAAAGACTGCCCTGACAATAGCATTAGTCATTCTGCTGAACCTAGTAAAGCTTCTGGTCCCCATAGCTCAAGTGGGACTAAATCCAATAAGAAAAAGGT

GAAGGTAGATTGGACACCTGAACTACACAAAAAGTTTGTTCAAGCAGTAGAGCAACTTGGTATAGATCAAGCCATTCCTTCTCGAATACTAGACGTGATGAAAGTGGAGGGCT

TAACGAGACATAACATAGCTAGCCATCTCCAGAAATACAGAATGCATCGGAGGCAAATTTTGCCAAGGGAAGTGGAAAGGAGATGGCCCCATCCGCAACCTAGAGATTCAGT

ACAAAGGAATTACTATCCTCATAAACCTGTCATGACATTCCCACCATATCATTCTAATCATGTCGCCCCAGCTGGTCAATGTTATCCTGCTTGGGTACCACCGGCTAGTTATCCGAA

TGGTTTACAAGTGTGGGGTTCACCTTACTATCCGGGATGGCAGCCTGCAGAGACTTGGCACTGGAAGCCTCATCCAGGGCTGCTTGCTGATACATGGGGCTCCCCTGTCATGC

CACCATCGTTTGGATCGTATCCACCATATCCCCAGAATGCTGGAATGTACCAGTCTCACGGAATGCATAACAGATATAGCATGCTAGAGAAGTCATTCGATGTTCACCCGGCAGA

GGAGGTGATTGATAAAGTAGTAAAGGAGGCAATAACCAAACCATGGTTACCACTTCCTTTGGGCTTAAAACCTCCTTCCATGGAGGGTGTACTCGACGAGCTTTCTAGACAAG

GAATCTCAACCGTCCCTCCACGAATCAATGGCTCTCGATGTTGGAGATGA

>AC08-045_Amino acid seq.

MICIEDELLGWKDFPKGLKVLLLDEDSNSAAEMKSRLEKMDYIVYTFCNESEALSAISSKSEGFHVAIVEVSAGDNDGVLQFLESAKNLPTIMTSNIHSLSTMMKCIALGAVEFLQKPL

SDDKLKNIWQHVVHKAFNARKDVSGPLEPVKESLLSMLQLQPEKGEPDDKSSNGTEPLIAVADNNTEQSSGCDKYPAPSTPQLKQGVRSVDDSDCHDHTIFSTDQDSGEHDGD

TKSVETTYNNSLAENTVQISPPGQQEDIILKEENGSSPHQTMEADIATFSQINDCADNSDGSSPHQKTEADIATTSSQSKDCPDNSISHSAEPSKASGPHSSSGTKSNKKKVKVDW

TPELHKKFVQAVEQLGIDQAIPSRILDVMKVEGLTRHNIASHLQKYRMHRRQILPREVERRWPHPQPRDSVQRNYYPHKPVMTFPPYHSNHVAPAGQCYPAWVPPASYPNGLQV

WGSPYYPGWQPAETWHWKPHPGLLADTWGSPVMPPSFGSYPPYPQNAGMYQSHGMHNRYSMLEKSFDVHPAEEVIDKVVKEAITKPWLPLPLGLKPPSMEGVLDELSRQGIS

TVPPRINGSRCWR-

>AC08-201_Amino acid seq.

MICIEDELLGWKDFPKGLKVLLLDEDSNSAAEMKSRLEKMDYIVYTFCNESEALSAISSKSEGFHVAIVEVSAGDNDGVLQFLESAKNLPTIMTSNIHSLSTMMKCIALGAVEFLQKPL

SDDKLKNIWQHVVHKAFNARKDVSGPLEPVKESLLSMLQLQPEKGEPDDKSSNGTEPLIAVADNNTEQSSGCDKYPAPSTPQLKQGVRSVDDSDCHDHTIFSADQDSGEHDGD

TKSVETTYNNSLAENTVQISPPGQQEDIILKEENGSSPHQTMEADIATFSQINDCADNSDGSSPHQKTEADIATTSS*SKDCPDNSISHSAEPSKASGPHSSSGTKSNKKKVKVDW

TPELHKKFVQAVEQLGIDQAIPSRILDVMKVEGLTRHNIASHLQKYRMHRRQILPREVERRWPHPQPRDSVQRNYYPHKPVMTFPPYHSNHVAPAGQCYPAWVPPASYPNGLQV

WGSPYYPGWQPAETWHWKPHPGLLADTWGSPVMPPSFGSYPPYPQNAGMYQSHGMHNRYSMLEKSFDVHPAEEVIDKVVKEAITKPWLPLPLGLKPPSMEGVLDELSRQGIS

TVPPRINGSRCWR-
