# Supplementary material for: Candidate Gene Analysis Reveals That the Fruit Color Locus C1 Corresponds to PRR2 in Pepper (Capsicum frutescens)
Source: Front Plant Sci. 2020 Apr 9;11:399. doi: 10.3389/fpls.2020.00399 (PMC7161348; doi:10.3389/fpls.2020.00399)
Supplement: Supplementary file 3 [file Presentation_1.pdf]

Supplementary table

Table S1. Carotenoid profiles of each accession.

Accession	Lutein	Neoxanthin	Antheraxanthin	Zeaxanthin	Total carotenoids (mg/100 g dW)
AC08-045	5.32±0.88 (0.88)	0.65±0.09 (0.11)	0.00±0.00 (0.00)	0.11±0.00 (0.02)	6.07±0.97
AC08-201	0.71±0.07 (0.70)	0.19±0.01 (0.19)	0.14±0.02 (0.14)	0.00±0.00 (0.00)	1.02±0.08

Supplementary figures

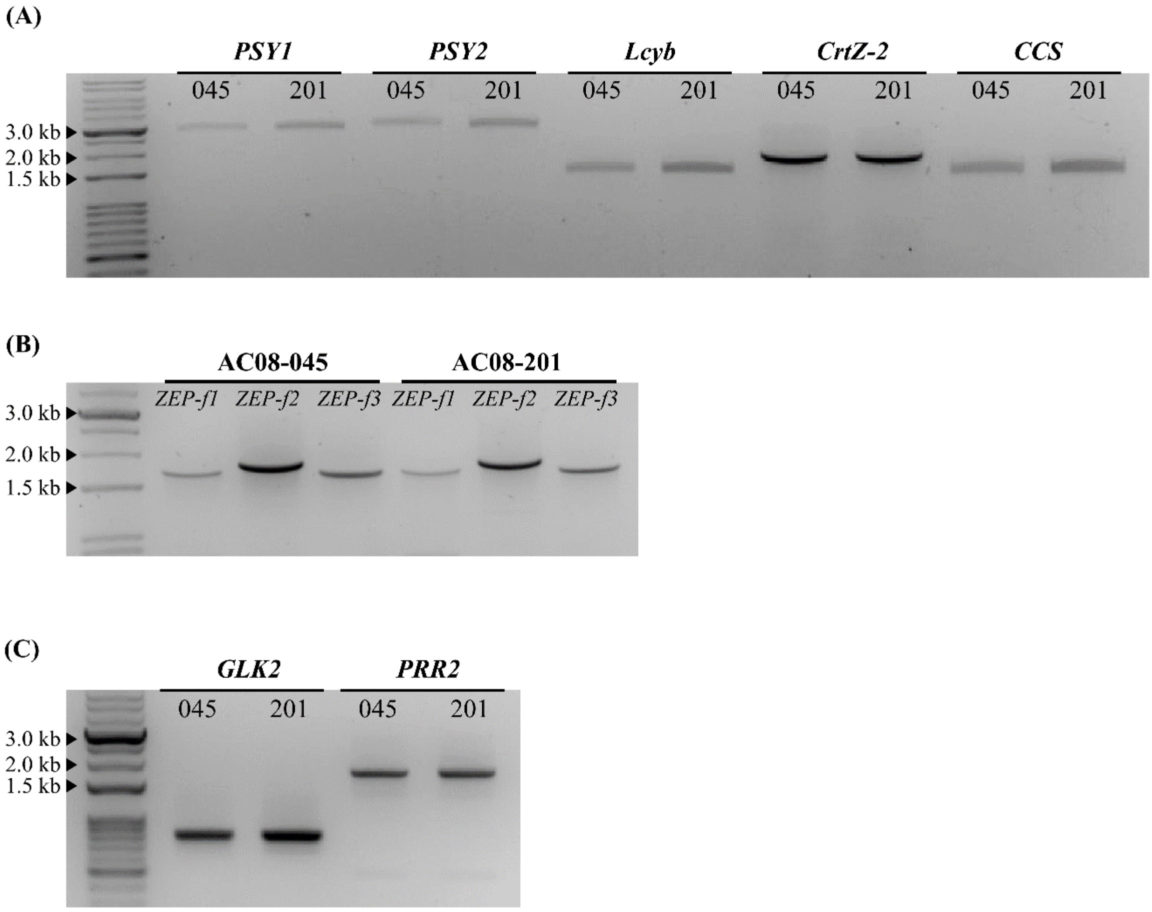

Fig. S1. PCR results of six carotenoid biosynthesis genes using gDNA and two plastid regulating genes using cDNA from AC08-045 and AC08-201.

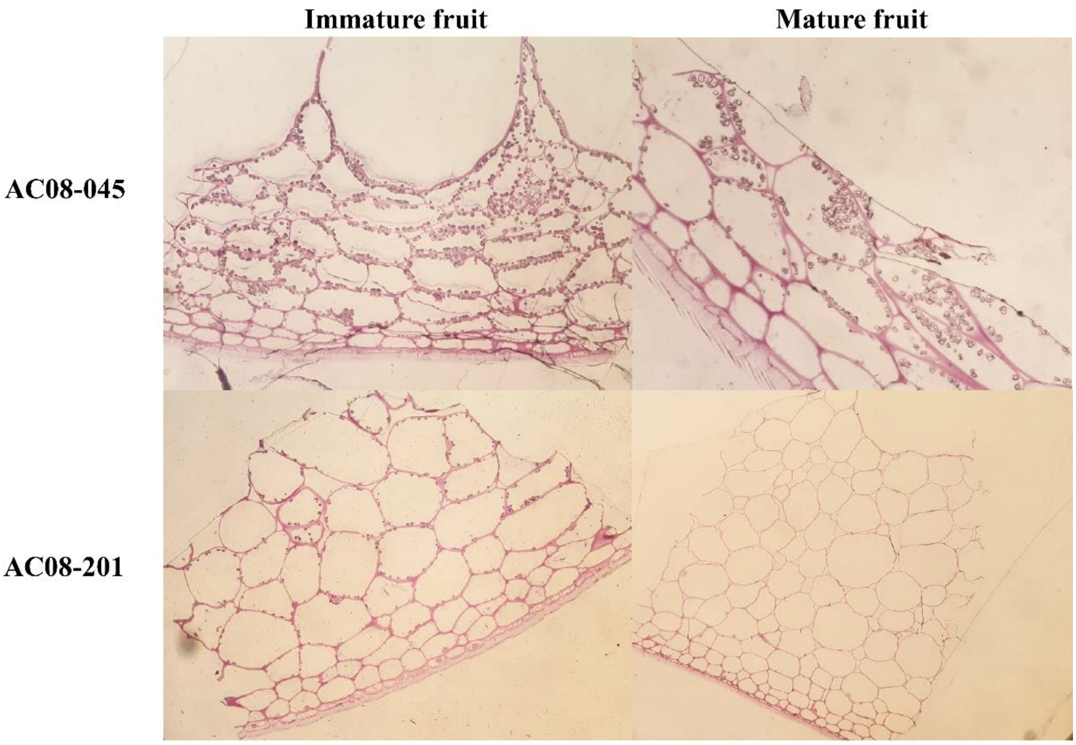

Fig. S2. Microscopic images of immature and mature fruit pericarps of AC08-045 and AC08-201 stained with toluene blue.

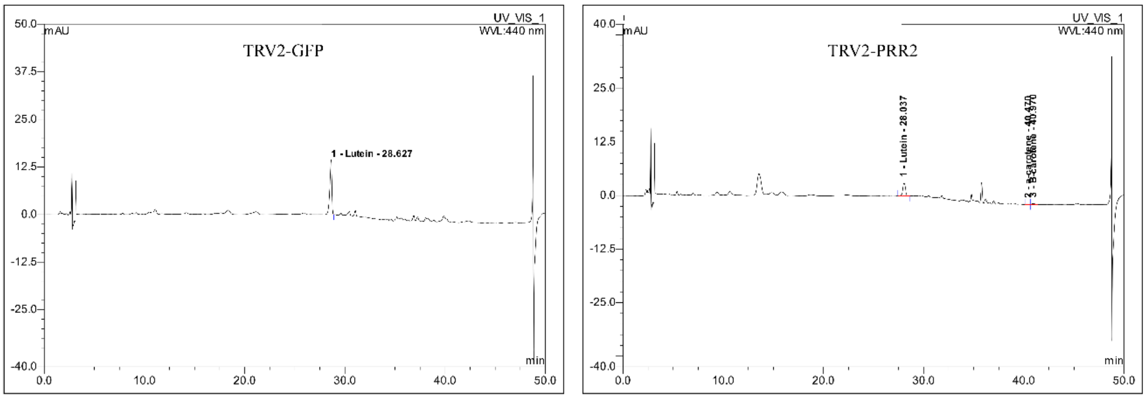

Fig. S3. HPLC profile of the mature fruits from MY plants subjected to VIGS.

Supplementary Materials

Supplementary Material 1. Alignment of coding sequence of *PSY1*, *CCS*, and *GLK2* between AC08 and CM334. (excel file)

Supplementary Material 2. Coding sequence and amino acid sequence of *PRR2* of AC08-045 and AC08-201. (pdf file)
